# Computational Optimization of Image-Based Reinforcement Learning for Robotics

**DOI:** 10.3390/s22197382

**Published:** 2022-09-28

**Authors:** Stefano Ferraro, Toon Van de Maele, Pietro Mazzaglia, Tim Verbelen, Bart Dhoedt

**Affiliations:** IDLab, Ghent University, 25843 Ghent, Belgium

**Keywords:** reinforcement learning, neural networks, post-training quantization, quantized-aware training, optimization

## Abstract

The robotics field has been deeply influenced by the advent of deep learning. In recent years, this trend has been characterized by the adoption of large, pretrained models for robotic use cases, which are not compatible with the computational hardware available in robotic systems. Moreover, such large, computationally intensive models impede the low-latency execution which is required for many closed-loop control systems. In this work, we propose different strategies for improving the computational efficiency of the deep-learning models adopted in reinforcement-learning (RL) scenarios. As a use-case project, we consider an image-based RL method on the synergy between push-and-grasp actions. As a first optimization step, we reduce the model architecture in complexity, by decreasing the number of layers and by altering the architecture structure. Second, we consider downscaling the input resolution to reduce the computational load. Finally, we perform weight quantization, where we compare post-training quantization and quantized-aware training. We benchmark the improvements introduced in each optimization by running a standard testing routine. We show that the optimization strategies introduced can improve the computational efficiency by around 300 times, while also slightly improving the functional performance of the system. In addition, we demonstrate closed-loop control behaviour on a real-world robot, while processing everything on a Jetson Xavier NX edge device.

## 1. Introduction

In recent years, reinforcement learning (RL) has gained increasing traction in robotics [1,2,3,4]. This rising popularity is in part attributable to the advances in the deep-learning field in general [5]. Thanks to this new control paradigm, a high level of robustness and flexibility can be achieved without the need to engineer complex heuristics for robotic systems [6,7]. Presently, deep learning is a solution adopted in a vast number of fields, becoming a standard approach for solving tasks that require a high level of generalization. The main trend in deep learning is to increase the number of model parameters, as well as the training dataset size, to outperform the state of the art on a vast variety of skills [3,8]. This trend does not always match the requirements of the field of robotics. Indeed, many robot systems are usually distributed systems, which have limited resources in terms of computational power, either due to the necessity to move around in the environment or due to the limit imposed by economic feasibility [9]. On the other hand, robotic systems interacting with a dynamic environment require a high feedback rate to be able to process all types of sensory information without being a bottleneck for the whole system [3,10]. Ideally, we would have a low-latency system controlled in a closed-loop fashion [7,11]. However, this contrasts with the trend in the deep-learning community to adopt larger and larger neural network (NN) architectures to process high-dimensional inputs such as RGBD data [3,12,13]. These architectures are often pretrained on a broad dataset and become a good starting point for every specific use case [12,13]. This reduces the overall training process, as only a few additional layers that are added to the pretrained architecture of choice need to be trained from scratch. However, the final performance of the system is directly related to the similarity between the type of task the pretrained system has been trained for [14]. In robotic use cases, where use-case scenarios are known and the environment is largely fixed, pretrained architectures are less beneficial, since each specific use case requires specific information (i.e., mastery of a specific skill, recognition of only specific types of objects). Indeed, for these systems, having a widely generalized architecture is not necessarily the most efficient option, and training a small NN from scratch might suffice [15]. An anthropomorphic analogy would be having the full potential of a human brain just to recognize some simple shapes, which would clearly be a waste of potential.

In this paper, we demonstrate that adopting a large pretrained NN architecture can actually be overkill for a typical RL scenario, and how, with a computationally optimized model, comparable performance can be reached. Such an optimized model will also result in a gain in processing latency, making the model at hand feasible for deployment on edge devices. Deployability on an edge device is of major interest for industrial applications where resources are often limited and where security issues arise from the upload of the raw sensory information to the cloud [16,17]. Major latency reduction will also open the model to a closed-loop integration in the system, making the system itself reactive to changes to the environment.

The main contribution of the paper is the raising of awareness in the adoption of pretrained models, demonstrating the potential of ad hoc models for specific robotic applications. In particular, we focus on an image-based RL method for a robot manipulator learning to push and grasp objects [12]. We propose different optimization strategies to reduce the computational load while keeping similar task performance.

1. First, we consider model reduction techniques such as the one adopted in MobileNet architectures severely decreasing in the number of layers and parameters of the model [18].
2. Second, we investigate the impact of input image resolution, as this parameter determines to a large extent the computational complexity both at training and inference times.
3. Third, we also explore quantization strategies as recently introduced approaches to decrease the computational effort of NN [19,20,21,22]. Quantization can be applied after the training process, i.e., post-training quantization, or during training, i.e., quantization-aware training.

**Figure 1:**
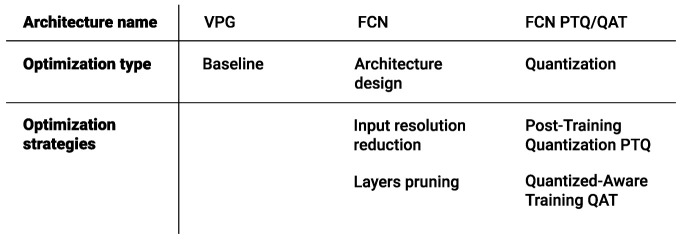
Overview of all the optimization strategies considered.

Overall, we show that the optimization strategies introduced can improve the computational efficiency by a factor of 300, without affecting the task performance. In addition, we demonstrate closed-loop control behaviour on a real-world Franka Panda robot, while processing everything on a Jetson Xavier NX edge device. An overview of all the optimization strategies considered in the paper are outlined in Figure 1.

In the next section, we first give an overview of the robot setup, the problem formulation and the original RL formulation and model architecture. Next, we present our optimized NN architectures, as well as different quantization methods considered. In Section 3, we extensively evaluate all considered architectures in a simulation environment, and we demonstrate our approach on a real-world robot setup.

## 2. Materials and Methods

We start from the original work from Zeng et al. [12], as a prototypical robotic RL use case. We first describe the general problem setup and Q-learning formulation, and then elaborate on our optimization steps for obtaining a more computationally efficient architecture than the original baseline.

### 2.1. Robotic Setups

**Figure 2:**
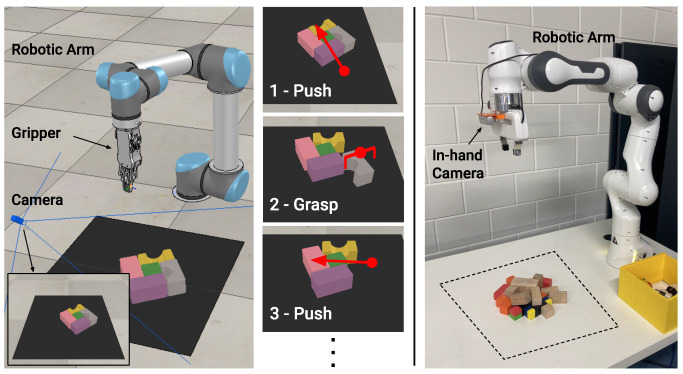
Robotic setups. On the left: the simulation environment consists of an UR5 robotic arm equipped with a two-finger gripper. An RGBD camera provides a side view of the workspace, which is delimited by the black square in front of the base of the robot. Objects are presented inside the workspace. On the right: th hardware environment consists of a Franka Panda robotic arm equipped with a two-finger gripper. An in-hand RGBD camera is used as a perception system. The workspace is software-limited to avoid collisions with the surrounding environment. Objects are presented inside the workspace. For both systems, the robotic arm in use must be able to grasp all the objects exploiting grasp-and-push actions.

The simulated setup consists of a simulated environment in CoppeliaSim [23], which is the same setup as used by [12]. As shown in Figure 2, it consists of a UR5 robotic arm, equipped with a two-finger RG2 gripper. The robot is presented with some objects, i.e., 7 different 3D shapes with randomly assigned colors, inside a limited workspace of $0.448\;m^{2}$ rendered as a black area in front of the robotic arm. The perception system consists of a side-view static RGBD camera.

Figure 2 shows our hardware setup, which consists of a Franka Panda robot arm equipped with an in-hand Intel Realsense D435 RGBD camera. Adopting an in-hand camera alleviates the limitations of the fixed, limited workspace of a side-view camera, and opens up a whole set of use cases with closed-loop control such as active vision [24]. The major implication in adopting a moving viewpoint is that the resulting action must be determined relative to the current camera position, in contrast to using a fixed-reference camera frame. A standard gripper for the Franka Panda is used. As in the simulated environment, the robot is presented with different shapes (four in total) with diverse colors. During training time, the workspace is software-limited and comprises a physical area of $0.448\;m^{2}$.

The goal of both the simulated and hardware systems is to grasp all the objects in the workspace. The same set of two primitive actions can be exploited by both robotic architectures: *Push* and *Grasp*. Both actions consist of two hard-coded routines, where both take as input the positional parameters *q*: center of action and $k_{d}$: orientation. For the push action, the parameter *q* represents the starting position of a 10 cm push, whose direction is given by parameter $k_{d}$. For the grasp primitive, *q* denotes the position of the end effector origin, $k_{d}$ instead represents the orientation of the end effector. Ultimately, each system must be able to synergistically use both actions to succeed in manually engineered scenarios such as the ones presented in Figure 5, where exploiting only grasping actions would result in suboptimal performance.

### 2.2. Problem Formulation

The push-and-grasp problem can be formalized as a classic reinforcement learning (RL) formulation, represented by a Markov Decision Process (MDP), denoted with the tuple {S,A,T,r,γ}, where S is the set of states, A is the set of actions, *T* is the state transition dynamics, *r* is the reward function, and γ is a discount factor. At each time step, the agent picks an action $a_{t}$ according to a policy $\pi (s_{t})$, a function of the current state $s_{t}$. As a result of the action $a_{t}$, the environment transitions to a new state $s_{t+1}$ according to the state transition dynamics, and provides the agent with a reward $r_{t}\left(s_{t},s_{t+1}\right)$ based on the difference between the two consecutive states. In our case, a state consists of RGBD data acquired by the camera after an action is completed.

The goal of the agent is to maximize the expected sum of future rewards $\sum_{i=t}^{\infty }\gamma^{i-t}r_{i}\left(s_{i},s_{i+1}\right)$, where γ∈[0,1) is a discount factor over an infinite horizon. It can do so by choosing actions that maximize the action-value function or Q-function $Q(s_{t},a_{t})$, which represents the expected return when choosing action $a_{t}$ in state $s_{t}$.

#### 2.2.1. Actions

As stated in Section 2.1, the set ψ of possible actions that the agent can perform are push and grasp. Positional parameter *q* is obtained by the projection of a pixel *p* to the workspace, and parameter $k_{d}$ is a degree value between 0 and 360. This yields us the following action space:

(1) $$a=\left(\psi ,q,k\right)|\psi \in \left\{push,grasp\right\},q↠p\in s_{t},k_{d}\in \left[0^{\circ },360^{\circ }\right]$$

Similar to [12], we use 16 possible rotations for the $k_{d}$ parameter, resulting in a discretization every $22.5^{\circ }$. The 16 discretized angles are indicated by the value of *k*, where k∈[0,15], corresponding to $\left[0^{\circ },22.5^{\circ }\cdots 337.5^{\circ }\right]$.

For the push action, the positional parameter *q* represents the starting position of a 10 cm push whose direction is given by parameter *k*. For the grasp primitive, *q* denotes the position of the end effector origin, *k* instead represents the orientation of the end effector. Ideally, if the geometric center of an object is placed at position $p_{l}$, the grasp primitive should be executed with $q\approx p_{l}$ and *k* along the shorter dimension of the object.

#### 2.2.2. Rewards

The system is provided with positive feedback after successful completion of an action. For a successful grasp, the agent receives a reward of +1. A grasp is considered successful if the width of the robot’s gripper, at completed grasping routine, is greater than zero. A successful push yields a reward of +0.5. The push action $a_{t}$ is considered successful if the difference in the depth maps between $s_{t}$ and $s_{t+1}$ is larger than a specific threshold. The interaction between push-and-grasp actions is not enforced in any way; the system must learn to predict the benefit of concatenating push-and-grasp actions by itself.

### 2.3. Processing Pipeline

**Figure 3:**
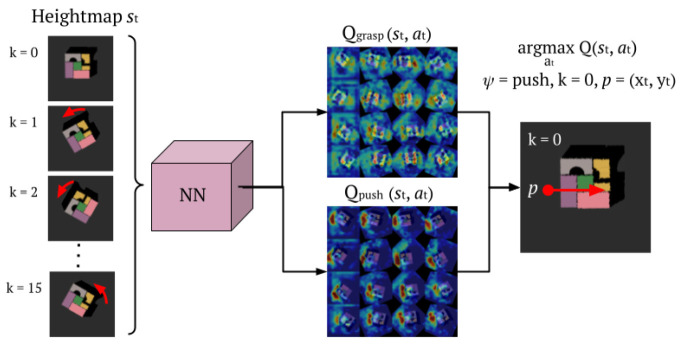
Data pipeline in the system. Each complete forward pass consists of 16 distinct forward passes through the NN. The input heightmaps (RGBD data) are rotations of the correspondent *k* value. The output of the system is 2 Q-value maps per each forward step, for a total of 32 maps. Out of these, the *k* map, of the action, presenting the maximum pixel Q-value identify the chosen action to perform.

We adopt a deep Q-Network (DQN) approach [25], in which the Q-function is approximated by a neural network. Here we focus on the processing pipeline, and details about each specific neural network implementation proposed in this work will be presented in Section 2.6. Figure 3 shows the processing pipeline of the system. The input is an RGBD heightmap of resolution of 640×640 px. The heightmap is obtained by an affine transformation of the original perspective captured by the camera sensor present in the setup in use. The edges of the heightmaps are aligned with respect to the boundaries of the agent’s workspace. To take into account the different rotations, the input is expanded and rotated *k* times, once for each rotation factor.

This input is then processed by the deep Q-network, which outputs two sets of Q-value maps, respectively, for the push and for the grasp actions. The resolution of each Q-value map is 20×20 px, which we up-sample to a 320×320 px resolution. Thus, the complete output consists of 32 Q-value maps, 16 of them representing the push Q-value maps, and 16 for the grasp ones. As mentioned in Section 2.2.1, every pixel *p* of the heightmap corresponds to a specific location *q* in the physical workspace. Each Q-value map hence represents the expected return for specific action at each specific location *q*. Out of the 32 Q-value maps, the pixel $p(x_{t},y_{t})$ with the maximum expected reward is picked, resulting in:

(2) $$\arg\max_{a_{t}}Q\left(s_{t},a_{t}\right)=\arg\max_{\psi ,k,p}Q_{\psi ,k}\left(p\left(x_{t},y_{t}\right)\right)$$

### 2.4. Training

As in [12], the deep Q-network is trained at each iteration *t* to estimate the state-value function using the Huber loss function:

(3) $$L_{t}=\mathrm{Hub}\left[Q^{\theta_{t}}\left(s_{t},a_{t}\right)-r_{t}\left(s_{t},s_{t+1}\right)-\gamma \max_{a_{t+1}}Q^{\theta_{t}^{-}}\left(s_{t+1},a_{t+1}\right)\right]$$

where $\theta_{t}$ are the parameters of the neural network at iteration *t*, and the target network parameters $\theta_{t}^{-}$ are held fixed between individual updates. We pass gradients only through the single-pixel *p* from which the value predictions of the executed action $a_{t}$ were computed. All other pixels at iteration *t* backpropagate with 0 loss.

We train by stochastic gradient descent (fixed learning rates of $10^{-4}$, momentum of 0.9, and weight decay $2e^{-5}$). To stabilize the training procedure, an experience replay buffer is implemented. We opted for prioritized experience replay [26] using stochastic rank-based prioritization for sampling. An exploration strategy is adopted to guarantee a balanced exploration of the entire action set ψ. We use ϵ-greedy exploration, with ϵ initialized at 0.5 then exponentially decreased over training to 0.1. Discount factor γ is set to 0.5.

When training on the simulated environment, initially 10 objects are spawned randomly in the central area of the workspace, and the robot is free to perform every action within the workspace. Once the workspace is empty, or the robot has performed 10 consecutive actions without receiving any reward, the environment is automatically reset and 10 new objects are spawned. Over training time, the spawn area reduces quadratically over iterations. On the one hand, during the initial part of the training, this exposes the agent to scenarios where the objects are farther apart, which is an ideal scenario for learning to perform both primitives separately. On the other hand, the decrease in spawn area later during training facilitates the exposure to scenarios where more objects are next to one another, requiring a synergy between push and grasp to be learned.

For training on the real-world setup, a set of 16 different objects are used. The setup is equipped with a bottom-less bin useful for an automatic reset. The environment is reset in the case of an empty workspace, or in the case that the robot has performed more than 10 consecutive unsuccessful actions and the number of points with a non-zero depth value is greater that a specific threshold. This strategy is adopted to avoid continuous resets of the system in the early stages of the system, while still helping the system to not become stuck in later stages of the training, were few remaining objects can disrupt the correct training trajectory.

### 2.5. Testing

**Figure 4:**
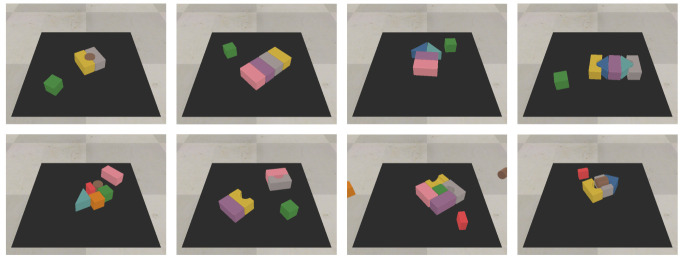
Examples of test scenarios for the simulated environment. Objects are tightly packed forcing the need for synergy between push-and-grasp primitives.

**Figure 5:**
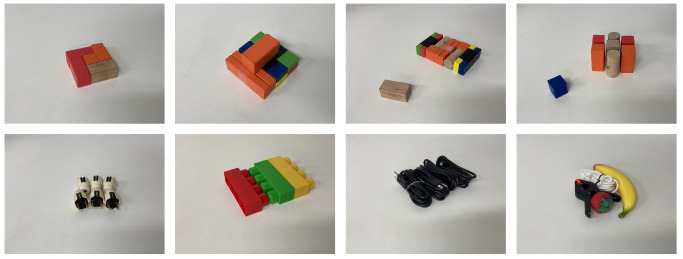
Examples of test scenarios for the real-world setup. Objects are tightly packed, forcing the need for synergy between push-and-grasp primitives. On the first row is a challenging composition made with toy blocks used in training. On the second row is a challenging composition made with novel objects.

The test set for the simulated environment consist of 10 hand-crafted challenging scenarios where a synergy between push and grasp is required to complete the task. Some of the scenarios are shown in Figure 4. For the real setup, similarly to the simulated one, we opted for a test set of six challenging hand-crafted scenarios made with the same objects used during the training procedure. In addition, the real robot setup is also tested on a second set of four challenging compositions made with novel, unseen objects. These scenarios are shown in Figure 5. Since our policies are greedy and deterministic during test time, it is possible that the agent becomes stuck repeatedly executing the same unsuccessful action, as no change is made to the environment. The original work [12] adopted a continuous training setting, where backpropagation is also performed at test time with a low learning rate, to continually integrate new information, and update the network. Due to the fact that we considered a static quantization method as a possible optimization strategy, which requires the architecture to be fixed, we opted for a different heuristic to solve this issue. In practice, we check if the same action $a_{t}$ is to be repeated consecutively at the same location with the same orientation, we select the second-best action. If the agent succeeds in grasping all the objects in the workspace, the scenario is considered successful. If any of the objects are pushed outside the workspace area, or the system performs 10 consecutive faulty actions, the scenario is considered failed.

### 2.6. Architectures

We compare several NN architectures to parameterize the deep Q-network. We start with the baseline of [12], and propose several optimizations thereafter.

#### 2.6.1. Baseline

**Figure 6:**
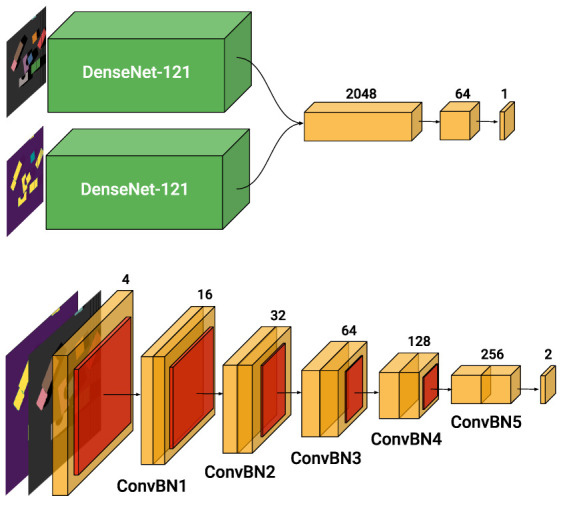
At the top: VPG architecture from the original work. Each ConvBN block represents a concatenation of a convolutional layer, followed by a batch normalization layer and a linear rectifier (ReLU). The input of the first DenseNet-121 architecture is RGB data, the second DenseNet-121 network receives as an input a cloned version of the depth data (DDD). The output consists of a single channel of 20 × 20 px that corresponds either to the push or the grasp Q-value map. The full system consists of two instances of the presented architecture, one for grasp primitives and the other for push primitives. At the bottom: Single-FCN architecture. Each ConvBN block represents a concatenation of a convolutional layer, followed by a batch normalization layer and a linear rectifier (ReLU). The input of the network is RGBD data, with a resolution of 640 × 640 px. The output consists of two channels of 20 × 20 px, which correspond to the push and grasp Q-value maps.

The original architecture proposed by [12], named VPG (Visual Pushing for Grasping), consists of two separate NNs, of which the first outputs the grasp values and the latter push values. The input of both networks is the heightmap of the workspace as an RGBD frame. Both the NNs have the same architecture shown in Figure 6, and they consist of two DenseNet-121 pretrained on Imagenet [27], followed by channel-wise concatenation and two additional 1×1 convolutional layers interleaved with nonlinear activation functions (ReLU) and spatial batch normalization [28]. One DenseNet receives as input the color (RGB) data, of resolution 640×640 px, while the second receives a cloned 3-channel depth (DDD) of the same resolution.

The baseline method consists of 32 million trainable parameters and requires 92 billion Multiply-ACcumulate (MAC) operations for processing a single image, which puts a big strain on resource use during both training and inference. For example, on an NVIDIA Titan X GPU, the mean average time for a single forward pass is around 150 ms, leading to an overall of 2.5 s considering the 16 rotations. Running the same architecture in an energy-efficient or cost-efficient device is not possible without a severe impact on both latency and memory requirements. Therefore, we redesigned the architecture. Instead of considering two separate NNs for the two distinct actions, we opted for a single architecture with two output channels for push and grasp, and avoided the use of large, pretrained DenseNet blocks.

#### 2.6.2. Single-FCN

We propose a single Fully Convolutional Network (FCN) for processing the RGBD inputs, as shown in Figure 6. Our network expects a single, four-channel tensor of RGBD data as input, avoiding data splitting. Furthermore, the architecture only consists of basic ConvBN building blocks, each made of a convolutional layer followed by batch normalization and a nonlinear activation function (ReLU). Two of these ConvBN blocks are grouped into five layers, named *ConvBN1*, *ConvBN2*, etc. At the start of every layer, a stride of 2 is used, halving the spatial resolution. This reduces the resolution from an initial 640×640 px of the input down to 20×20 px. The output is then up-sampled to be mapped back to a coordinate *q* in the workspace. At the start of each layer, we stride and hence reduce the spatial resolution, and double the number of channels. The maximum depth of the system is 256 channels. The kernel size used in the convolution layers is parameterized with respect to the input resolution of each layer, i.e., for a resolution greater than 160×160 px, it is fixed at 5×5, for all the lower ones at 3×3. The architecture results in a total of 1.3 million parameters and around 3.5 billion MAC operations, 25 times fewer parameters and 26 times fewer MAC operations with respect to the baseline method.

### 2.7. Input Resolution

A second optimization strategy that we considered is the reduction of the input resolution to the network. Different resolutions have been tested to a minimum of 80×80 px. The striding applied at each convolutional layer has been adapted to always have an overall output of resolution 20×20 px. We also considered variations in the number of block layers, starting from the complete configuration shown in Figure 6. We did some ablation studies removing the first layers *ConvBN1* and *ConvBN2*. We refer to the ablated architecture without layer *ConvBN1* as *4L*, and for the one lacking both *ConvBN1* and *ConvBN2* as *3L*. The original architecture of single-FCN corresponds then to *5L*. Throughout the manuscript, we name each architecture based on the input resolution and the number of major layers present in the architecture (e.g., the proposed architecture shown in Figure 6, will be named *FCN 640-5L*).

Table 1 gives an overview of the number of MACs required by each architecture, while varying both the input resolution and the number of layer blocks. It is clear that those parameters have a big impact on the number of MACs. Please note that given the same input resolution, we have a higher number of MACs when decreasing the number of layers in the model. Although this might seem counterintuitive, this is the consequence of a higher-resolution space applied to a higher number of channels (e.g., when considering architecture 4L, the initial resolution of 320 is applied directly to 32 channels, instead of 16 for the original 5L structure.)

### 2.8. Quantization

Quantizing weights down to int8 (8-bit integer, int8) values, has several advantages. When moving from 32 to 8 bits, the memory required to store the tensors decreases by a factor of four while the computational cost for matrix multiplication reduces quadratically by a factor of 16 [19]. Of course, this comes at the cost of a reduced precision, which might affect task performance. We consider two quantization methods: one where we quantize the NN weights post hoc using post-training quantization, and one where we take into account the quantization of the weights during training.

#### 2.8.1. Post-Training Quantization

Post-training quantization (PTQ) takes a trained full-precision model (32-bit floating point, fp32) and quantizes (to an 8-bit representation, int8) its weights to lower-precision values [19]. Uniform affine quantization is used to transform the fp32 values into int8 ones [29]. The process consists of first extracting the maximum and minimum values in the fp32 working range. The obtained range *r* is divided by $2^{n}$ to obtain the *scale factors*, with *n* the number of bits we are quantizing to, i.e., for an int8 quantization n=8 yields $2^{8}-1=255$ intervals. A parameter known as *zero-pointz* is used to ensure that the real zero value is quantized properly. Once the quantization parameters are defined, we can proceed with the quantization process. Starting from a floating-point value *x*, we map it to the unsigned integer grid $\left\{0,\cdots ,2^{n}-1\right\}$:

(4) $$x_{int}=clamp\left(\left\lfloor \frac{x}{s}\right\rceil +z;0,2^{n}-1\right)$$

where ⌊.⌉ is the rounding operator to the nearest integer and the clamp function is defined as:

(5) $$clamp\left(x;a,c\right)=\left\{\begin{array}{cc} a, & x<a\\ x, & a\leq x\leq c\\ c, & x>c \end{array}\right.$$

We can perform a *de-quantization* step [19] to obtain the approximate value of *x* in the original floating-point notation by:

(6) $$\hat{x}=s\left(x_{int}-z\right)$$

Any value of *x* outside the range *r* is clipped to *b* or *c* accordingly, which is known as *clipping error*. Increasing the range *r* will reduce the amount of *clipping errors*. However, increasing the range *r* also leads to an increase in *rounding errors*, i.e., the difference between $\hat{x}$ and *x*.

This quantization method is well suited for use cases where the working range of values is limited and fixed [19]. Moreover, the rounding error must be sufficiently low to guarantee a flawless conversion process. For our application, we opted for the *quantize* package provided in PyTorch [30]. The quantization process consists of three main passages:

1. *Fusing*; Since we are considering a static model, it is beneficial to fold the batch normalization layers into the convolutional layer. Besides reducing the computational overhead of the additional scaling and offset, this prevents extra data movement.
2. *Calibration*; The network is fed with random samples from the training dataset, for which we track the maximum and minimum values both at weight level and activation level. These values are instrumental for determining the maximum and minimum of the range *r*.
3. *Deployment*; The weights of the model are finally quantized. At every forward pass, the input of the model is quantized and then fed to the network. The output of the model is then de-quantized to the initial floating-point notation.

#### 2.8.2. Quantization-Aware Training

A last type of optimization that we consider is quantized-aware training (QAT). In contrast to post-training quantization, we now directly train a quantized model. Training using a quantized model helps to mitigate the approximation introduced by the quantization process, thus generally resulting in better performance when compared with PTQ methods [22]. We now start with a pretrained float32 model and further fine-tune, to improve the obtained performance. As most of the hardware adopted for the training process only supports floating-point representation, we insert so-called “fake” quantization blocks in the model during training. These quantization blocks produce a floating-point representation of the int8 quantized weight and activation values, and allow for backpropagation.

Backpropagating through a quantized model raises a major issue since the gradient of round-to-nearest operation in Equation (4) results in either zero or an undefined value, which translates to the impossibility to train the model [19]. A solution to this issue comes from the adoption of straight-through estimator (STE) [31]. Using this approximation, we can now compute the derivative of Equation (5), which results in:

(7) $$\frac{\partial \hat{x}}{\partial x}=\left\{\begin{array}{cc} 1, & a\leq x\leq c\\ 0, & otherwise \end{array}\right.$$

Using this gradient, we can now backpropagate though the quantization block.

### 2.9. Closed-Loop Control

To demonstrate the potential of an optimized NN model, we deployed a closed-loop pipeline based on the trained optimized models. For this, we use our real-world setup, with the processing being executed on a Jetson Xavier NX from NVIDIA. In terms of performance, this dedicated hardware is in line with common edge devices that can be found also in industry. It maintains low energy consumption, while being optimized for inference on a GPU architecture. For NN inference, we use hardware acceleration using the tensorRT back end from NVIDIA, which also supports int8 representations.

The robotic arm is now moved by a Cartesian velocity controller, with the target velocity proportional to the current distance of the end effector with respect to the target pose. The closed-loop pipeline continuously updates the target pose based on the best graspable pose provided by model inference of the latest camera data. In addition, the low-level control software smooths the raw output signals, to avoid discontinuities in the control signal. In this case, when the system is presented with a single object that is manually moved by a human operator, the robot arm will reach for the object and follow along with the operator movement, resulting in a more intuitive and flexible human–robot interaction. This type of control strategy requires high refresh rate for the control signal, as a low refresh rate would result in jerky movements, non-ideal for the mechanical maintenance of the robotic arm and in general not a save condition for collaborative robots.

## 3. Results

We performed a series of experiments to test the performance of the different architectures proposed. The goal of the experiments is to show: (i) preservation of the grasp-and-push accuracy reached by the baseline method; (ii) preservation of performance in testing scenarios, where synergy is required to solve the proposed human-engineered scenarios; and (iii) the reduction in computation time due to our optimizations.

### 3.1. Metrics

We test all architectures after training by presenting both the simulated and real system with challenging human-engineered scenarios that have not been seen during training time, as mentioned in Section 2.5. For the simulated system, each test scenario is performed n=5 times. Performance metrics are (1) the average % completion rate over the *n* test runs, which measures the ability of the policy to finish the task by picking up all objects without failing consecutively for more than 10 attempts, and without pushing any of the objects outside the workspace area; (2) the average % grasp success rate per completion; and (3) the % action efficiency, defined as $\frac{\#\;\mathrm{objects}\;\mathrm{in}\;\mathrm{test}\;\mathrm{scenario}}{\#\;\mathrm{action}\;\mathrm{before}\;\mathrm{completion}}$, which describes how readily the policy in use is able to solve the problem at hand, without performing unnecessary actions (e.g., multiple pushes). For all the metrics considered, a higher value is better. For the real-world setup, each scenario is presented once. Performance metrics are the same considered for the simulated environment. In conjunction with these metrics, the computational effort is evaluated by measuring the forward time (i.e., the time required for the input data to be processed by the network) on a standard hardware platform.

### 3.2. Training

**Figure 7:**
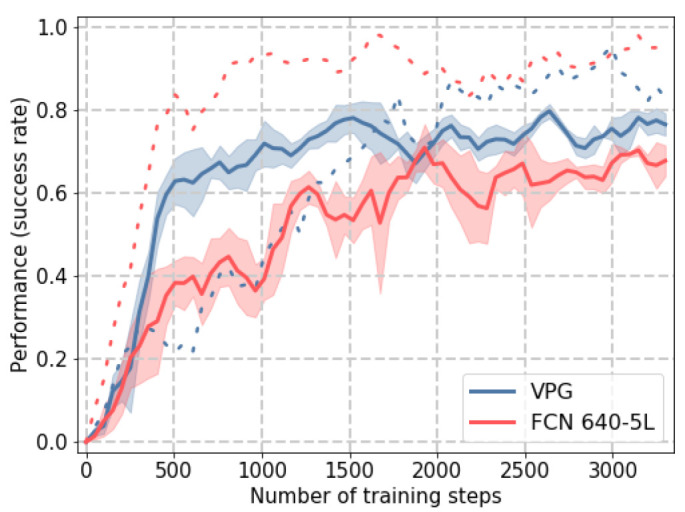
Comparison in training performance between the baseline architecture VPG (based on the DenseNet-121 architecture) and the proposed single-FCN with an input resolution of 640 × 640 px in the simulation environment. The solid line represents the grasping performance (success rate over the previous 200 iterations), while the dashed line represents the push performance.

Before looking at test performance, we compare the evolution of the grasp-and-push success rate as training progresses. In the simulation environment, we train both the baseline VPG and FCN-5L with 10 objects, and average the performance over three random seeds. Figure 7 shows the performance during training, and both VPG and FCN 640-5L reach a similar level of grasp accuracy at convergence. The push action (dashed line) is learned faster by the FCN 640-5L, but again similar levels are reached by both architectures at the end of training. For the grasp action, the VPG has a steeper learning curve, reaching a maximum value of grasp accuracy that is slightly higher than the FCN 640-5L. Please note that the average forward time of the baseline architecture is around 35 times the FCN 640-5L one, which leads to a significant speedup of the training process.

### 3.3. Testing

Here, we present the results of the testing scenarios for both the simulation and the real-world setup.

#### Simulation Environment

**Figure 8:**
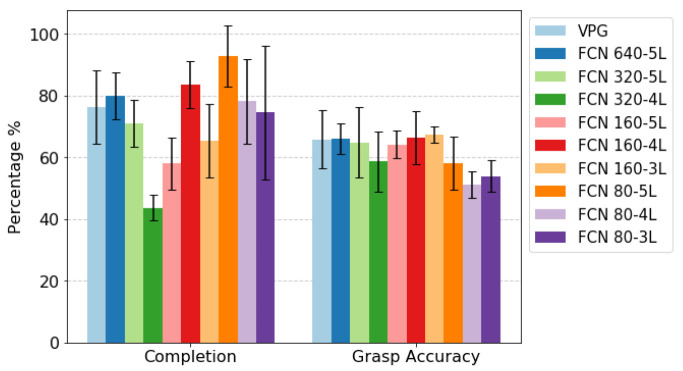
Comparison over testing scenarios between the proposed architectures. Error bars represent the standard deviation over five distinct runs of the testing procedure.

We benchmark the results of the proposed architectures on their performance on the simulated test scenarios. Results are shown in Figure 8. Despite the large differences in number of parameters and MAC operations, there are no significant differences in terms of grasp accuracy. Only when scaling down the input resolution to 80×80 px do we witness a slight decrease in performance, probably due to the higher loss of information about key features of the image. In terms of completion performance, the differences are more apparent, but there is no clear pattern. We notice how the FCN 640-5L architecture performs slightly better compared to baseline, which does not coincide with the success rates reached at the end of the training process shown in Figure 7. This illustrates that there are more subtle factors to consider than just the grasping performance for the overall performance of the system. By observing how these different policies behave in the different scenarios, it is evident that although a specific policy has learned to grasp isolated objects with a high success rate, and at the same time also to successfully push clusters of objects, when exposed to a test scenario, the key factor is the balance between the two. This means that the decision process has not been optimized for the long horizon yet, and that synergies between actions are not learned properly. For example, referring to policies that had a poor completion rate, we noticed how these tend to prefer push actions also on isolated objects, leading to an increased probability of pushing these outside the workspace area. Jointly considering both completion and grasp accuracy performance, architecture FCN 160-4L is the one that overall gave the best performance.

We also investigated the performance after static quantization. As a target architecture for the PTQ process, we opted for the FCN 160-4L. As introduced in Section 2.8.1, after the first step of fusing the convolutional layers with the normalization ones, we proceed with the calibration step, using 1000 RGBD images taken randomly from the whole training dataset. As shown in Table 2, both systems perform similarly. We can conclude that the approximations introduced by the quantization process do not have a significant impact on either grasping or pushing performance, nor the synergy between the two.

### 3.4. Computation Time

**Figure 9:**
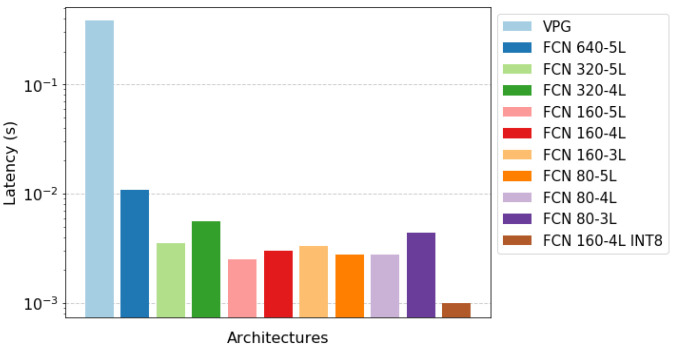
Comparison over forward time of the proposed architectures.

We benchmark the proposed architectures based on the inference time. We opted for an NVIDIA Jetson Xavier NX board since in terms of computational power it resembles a device that can be found as an edge device on industrial machinery [9]. Each test has been run exploiting hardware acceleration provided by the Jetson board. In particular, we used the TensorRT library as software interface to the accelerators, which provides both accelerator interfaces for FP32 and INT8. Since the quantization method (PTQ or QAT) did not impact the latency, we just denote the quantized architecture as FCN 160-4L INT8. The results are shown in Figure 9. The inference time considers a single forward pass through the neural network, but during a complete execution step the system forward *k* times through the network, where *k* corresponds to the number of orientations considered, in our case k=16. This results in a complete forward time of about 6.10 s for the VPG architecture, compared to 0.17 s for FCN 640-5L. After quantization, a minimum of 0.016 s is obtained, which corresponds to 62 Hz. For comparison, the maximum frequency observable by the human eye is 60 Hz.

#### Real Environment

For the real robot setup, we opted for architecture FCN 160-4L, due to the best performance reached during the simulation testing. We trained the proposed architecture on the real robot for 2000 training steps, on a set of 16 objects randomly placed. In addition, we also test the PTQ version of this model. As introduced in Section 2.8.1, in this case we first fused the convolutional layers with the normalization ones, after which we performed the calibration step using 1000 RGBD images taken randomly from the complete real robot training dataset. Finally, we also trained a third model with QAT. As mentioned in Section 2.8.2, it is advised to start from a pretrained model. We opted for the FCN 160-4L model previously trained in the real robot and further fine-tuned for 200 training steps.

We tested the real-world setup with each of the three models described above for challenging scenarios as shown in Figure 5. The results on the set of scenarios with training objects are presented in Table 3.

Best performance in terms of both grasping accuracy and action efficiency are reached by the PTQ model. This is surprising since a static quantization process is typically expected to deteriorate the performance of the native model [19].

In addition, we also tested scenarios with novel, unseen objects. Results for this testing regime are presented in Table 4. Overall, the system has some good generalization properties to novel objects. The synergy between push and grasp is also exploited with novel objects. As a demonstration, we provided some videos as Supplementary Materials.

### 3.5. Closed Loop

Finally, we performed a qualitative test on the closed-loop system. Again, starting from the pretrained FCN 160-4L model we quantized it using a static approach. The quantization is carried out using torch2trt library. Torch2trt is a converter for PyTorch models to a TensorRT model. In the conversion process, different precision formats can be adopted. As for the PTQ process carried out using the PyTorch Quantization module, a calibration process is required. We opted for a dataset of 500 training images for this calibration process.

Considering the whole processing pipeline, described in Section 2.3, necessary for achieving both the best grasping pose and rotation setting (over 16 levels of discretization), we reach an average of 22.3 FPS (Frames Per Second) in the overall processing. This rate of update leads to a smooth closed-loop control. If we would consider the original fp32 FCN 160-4L model, performance overall would drop to an average of 10.2 FPS. For comparison if we would consider the initial baseline, performance would drop to 0.2 FPS.

A video of the closed-loop system is available in the Supplementary Materials, which demonstrates the performance achieved by the quantized system.

## 4. Discussion

In this paper, we presented different optimization strategies for a robotic manipulator RL use case. First, we significantly reduced the complexity of the model proposed by [12] by avoiding bloated, pretrained models and sharing layers for different action modalities. Second, we proposed some further optimizations by lowering the input resolution to the system and by varying the number of convolutional layers. Finally, we opted for a post-training quantization method on the best-performing proposed architecture. When comparing the baseline method to the final quantized model, we have an improvement, in terms of computational performance, of around 300 times. This is without affecting the performance of the system’s functional metrics. We did not find a significant gain in performance using quantized-aware training (QAT) compared to post hoc quantization. Additional research is required to further quantify the impact of quantization-aware training in a reinforcement-learning context.

Our work shows that, despite the huge potential of adopting deep-learning techniques, and especially in the context of deep-reinforcement learning for robotics, care needs to be taken when designing and evaluating such systems. Notwithstanding the merit of pretrained models, it must be taken in consideration whether such a model is the best suited architecture for a given use case. Additionally, the obtained rewards of an agent are not necessarily reflective of its performance in particular test scenarios. Finally, a two orders of magnitude speedup can be achieved by optimizing and quantizing the model, which is crucial for enabling closed-loop control systems.

As our work targets the robotics use case of pushing and grasping in a reinforcement-learning setting, this will prove useful in various applications, such as pick and place, bin emptying, or product sorting. Introducing a push actuation can improve the overall efficiency of the system by reducing the number of faulty grasp attempts. In addition, closed-loop control is of utmost important for robotic systems operating in dynamic environments, where objects can move during interaction. Especially in human–robot collaboration scenarios, it is crucial that the robot can sense and react to the human’s actions at a very low latency. Moreover, using the optimization methods introduced opens the door for more affordable and energy-efficient edge devices for inference.

In this work, we mainly focused on the computational optimization of an RL system. It would be of interest for further work to extend the current work with the optimization aspect on the converge timing for the training process of such system. In particular, a relationship between the decrease for converge timing and the computational performance would be of interest. Simulation to reality (sim2real) methods would be the main focus of the proposed track [32,33]. Possible lines of experimentation would be related to limiting the data pipeline to either RGB or depth data, to measure the impact of the sim-to-real data processing for both data streams (RGB and depth) independently.

## Figures and Tables

**Table 1:** Number of MAC operations for each individual architecture considered.

Resolution	5 Layer Blocks (5L)	4 Layer Blocks (4L)	3 Layer Blocks (3L)
**640 × 640**	3.49 G	-	-
**320 × 320**	1.14 G	2.43 G	-
**160 × 160**	616.66 M	872.66 M	1.4 G
**80 × 80**	532.18 M	550.1 M	616.66 M

**Table 2:** Testing results for quantized system on a simulated environment.

Methods	Grasp Accuracy %	Action Efficiency %	Completion %
**FCN 160-4L**	66.4 ± 8.5	47.3 ± 5.2	83.6 ± 7.6
**FCN 160-4L PTQ**	65.3 ± 2.9	51.4 ± 6.5	85.4 ± 10.4

**Table 3:** Results for real robot setup tested on toy blocks challenging scenarios. Optimized 160-4L architecture is considered. Both Post-Training Quantization (PTQ) and Quantized-Aware Training (QAT) are explored.

Methods	Grasp Accuracy %	Action Efficiency %	Completion %
**FCN 160-4L**	56.4	50.0	100.0
**FCN 160-4L PTQ**	62.0	58.3	100.0
**FCN 160-4L QAT**	57.2	49.3	100.0

**Table 4:** Results for real robot setup tested on novel objects challenging scenarios.

Methods	Grasp Accuracy %	Action Efficiency %	Completion %
**FCN 160-4L**	61.5	50.0	100.0
**FCN 160-4L PTQ**	63.9	56.8	100.0
**FCN 160-4L QAT**	64.9	53.2	100.0

## Data Availability

The raw data supporting the conclusions of this article will be made available by the authors, without undue reservation.

## Supplementary Materials

The following supporting information can be downloaded at: https://www.mdpi.com/article/10.3390/s22197382/s1.
